# A cut above: atypical proteolysis endows complement C3 with non-canonical immune activities

**DOI:** 10.1038/s44318-025-00633-8

**Published:** 2025-11-17

**Authors:** Erin E West, Claudia Kemper

**Affiliations:** Complement and Inflammation Research Section, National Heart, Lung, and Blood Institute (NHLBI), National Institutes of health (NIH), Bethesda, MD 20892 USA

**Keywords:** Immunology, Post-translational Modifications & Proteolysis

## Abstract

A recent study identifies a novel proteolytic fragment of C3 that modulates complement activation and IL6ST signalling and correlates with autoimmune disease regression.

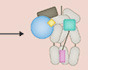

## Teaser

Well-defined proteolysis of complement component 3 (C3) produces fragments that mediate its classic activities including target opsonization and phagocytosis. In this issue of The EMBO Journal, Demir et al, (2025) uncover a novel C3 fragment, C3-LHF1, in the plasma of individuals with systemic lupus erythematosus (SLE), that curbs complement activation, modulates IL6ST signalling, and correlates with disease regression.

Long considered a simple degradative process driving protein turnover, targeted and limited proteolysis is now recognized as a fundamental physiological mechanism that orchestrates the activation, maturation, and termination of immune responses (Klein et al, 2018). Controlled proteolytic cleavage can profoundly alter the stability, localization, and biological activity of proteins, thereby ensuring spatial and temporal precision in immune signaling. Consequently, perturbations of proteolytic pathways contribute to immune pathologies, such as chronic inflammation, autoimmunity, cancer, and impaired host defense, overall making proteases attractive therapeutic targets. Profiling of protein N termini enables identification of distinct proteoforms that arise from alternative translational start sites, posttranslational modifications, or limited proteolysis. Recent advances in proteome profiling, (neo)N termini enrichment, and high-throughput automation have now made comprehensive analysis of proteolysis, even from samples with low protein content or rare patient cohorts, broadly accessible (Uzozie et al, 2022).

In the current issue of *The EMBO Journal*, Demir et al (2025) report an in-depth analysis of plasma N-terminomes from 143 individuals with systemic lupus erythematosus (SLE). By linking N termini signatures to clinical parameters, they identify a previously undescribed, biologically active fragment derived from the complement key component C3 that may associate with disease remission. As a first step, the authors optimized retrieval of N termini from human plasma by refining the established High-efficiency Undecanal-based N Termini EnRichment (HUNTER) protocol (Uzozie et al, 2022). Their modifications improved enrichment depth and purity, as demonstrated through benchmarking against the original method and a proof-of-principle experiment in which pharmacological inhibition of a proteolytic event produced the expected alterations in N termini profiles. Applying their optimized workflow (termed SHUNTER; Demir et al, 2025) to plasma samples from a well-characterized observational cohort with heterogeneous SLE phenotypes, they mapped over 11,000 protein N termini and revealed distinct, disease-linked proteolytic patterns. Multi-omics factor analysis (MOFA) integrated these signatures with clinical parameters, such as renal function, inflammation, and treatment response. In line with the established role of uncontrolled complement activation as a driver of persistent inflammation and tissue injury in SLE (Weinstein et al, 2021), alterations in N termini profiles were particularly prominent for complement proteins, especially for C3, compared with healthy donor plasma.

**Figure 1:**
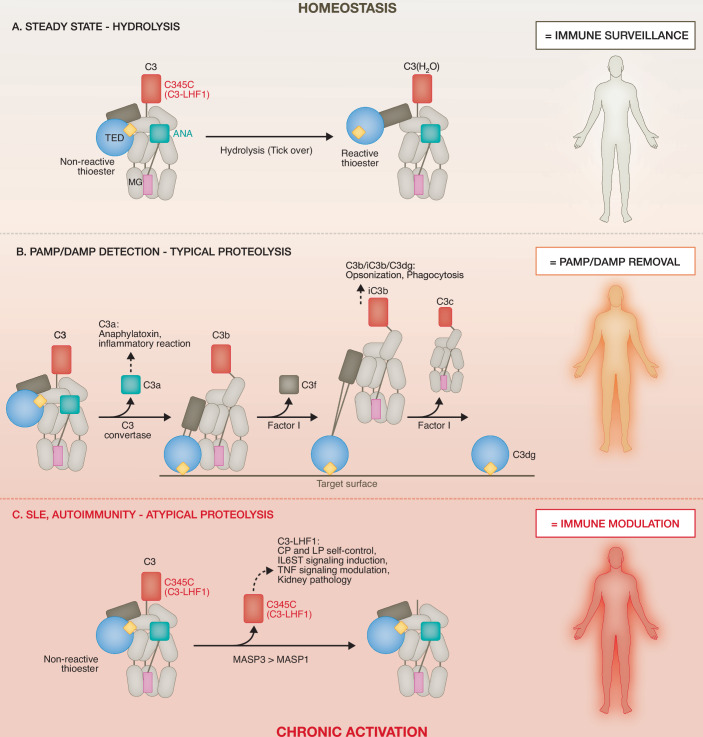
Atypical proteolysis of complement C3 in SLE creates activation fragments with noncanonical immune activities. (**A**) Simplified schematic of C3 and the hydrolysis-activated immune-surveilling C3(H_2_O) form with key functional domains highlighted. Gray ovals denote macroglobulin (MG) structural backbone/support domains. (**B**) Detection of pathogenic or self-derived danger triggers classical proteolytic activation of C3 and generation of diverse C3 fragments with noted canonical activities principally aimed at danger removal. (**C**) Plasma C3 in individuals with SLE undergoes atypical proteolysis by MASPs 1 and/or 3. The released C3-LHF1 domain may negatively control complement CP and LPs activation, but also induce IL6ST signaling and podocyte loss/kidney injury. ANA anaphylatoxin domain, C345C most *N*-terminal domain of C3 (denoted also C3-LHF1), CP classical pathway, DAMP danger-associated molecular pattern, IL6ST IL-6 signal transducer (gp130), LP lectin pathway, PAMP pathogen-associated molecular pattern, SLE systemic lupus erythematosus, TED thioester-containing domain (thioester, yellow rhombus), TNF tumor necrosis factor.

The complement system exemplifies an immune effector network that depends on precision proteolysis for its activity (Mastellos et al, 2023). This ~50-protein-member-strong system surveys blood, lymph, and interstitial fluids through pattern-recognition molecules that circulate predominantly as inactive precursors requiring proteolytic activation. Upon sensing pathogens or danger-associated self-antigens, the classical, lectin, and alternative complement activation pathways converge on C3, the pivotal component of complement biology. C3 consists of two multidomain chains linked by a disulfide bond and undergoes major structural rearrangements upon targeted cleavage (Janssen et al, 2005) (Fig. 1). A small fraction of plasma circulating C3 undergoes spontaneous hydrolysis by water, exposing a reactive thioester that enables covalent attachment to activating surfaces such as microbes, thereby contributing to immune surveillance even at homeostasis (Fig. 1). Controlled proteolytic conversion of C3 through C3 convertases generates defined fragments with well-characterized effector functions: the anaphylatoxin C3a promotes immune cell recruitment and activation, while C3b opsonizes targets for phagocytic uptake by scavenger cells (Mastellos et al, 2023). Further proteolysis of C3b to iC3b and C3dg facilitates clearance of immune complexes and apoptotic material. Because excessive C3 activation underlies many inflammatory conditions, modulation of C3 cleavage and its fragments represents a major therapeutic avenue (West et al, 2024).

Unexpectedly, Demir et al, (2025) identified a novel C3 fragment encompassing the C-terminal C345C domain, cleaved at position 1514, which they termed C3-LHF1 (Fig. 1). Remarkably, C3-LHF1 was present at levels comparable to canonical fragments such as C3a and C3b-c in both healthy and SLE plasma. Its stability was confirmed using dietary pulsed stable-isotope (^13^C_6_-lysine) labeling in mice, which also revealed a murine orthologue of the fragment. Structural and size-exclusion chromatography analyses of recombinantly expressed human C3-LHF1 demonstrated that it folds into the expected C345C domain architecture. An in vitro protease substrate screen identified the lectin pathway serine proteases, mannan-binding lectin serine protease (MASP)1 and MASP3, as enzymes capable of generating C3-LHF1 through specific cleavage of C3. To begin exploring the biological relevance of C3-LHF1, the authors examined whether its generation correlated with molecular and/or clinical features in their SLE cohort. Indeed, C3-LHF1 abundance is associated with three MOFA factor modules linked to kidney-related phenotypic variations. Moreover, N termini profiling of plasma from six patients with lupus nephritis (a severe manifestation of SLE and important predictor of morbidity (Weinstein et al, 2021)), before and after remission-inducing therapy, revealed a marked decline in C3-LHF1 upon remission. Together, these observations suggest that C3-LHF1 generation mirrors disease activity and clinical outcome in SLE.

Functional experiments revealed that C3-LHF1 effectively inhibited activation of the classical and, most notably, the lectin pathway, while having minimal effect on the alternative pathway. Although the molecular mechanism remains to be defined, current data indicate that C3-LHF1 may interfere with MASP1/3 enzymatic activity or with the formation and/or stability of C3/C5 convertases. Unexpectedly, the biological reach of C3-LHF1 extended beyond complement. When added to cultured cell lines, it altered cell morphology, prompting pull-down assays using C3-LHF1 as bait and human kidney lysates as a protein source. This strategy identified the interleukin-6 signal transducer (IL6ST, gp130, and CD130) as a potential C3-LHF1-interacting partner. IL6ST forms, together with IL-6Rα, the IL-6 receptor complex (Osman and Neamati, 2024). Functionally, C3-LHF1 acted as a partial gp130/IL6ST agonist, initiating IL6ST-dependent JAK–STAT3 signaling and phosphorylation of downstream targets in human kidney organoids. Strikingly, these effects occurred independently of IL-6Rα. Furthermore, C3-LHF1 potentiated tumor necrosis factor (TNF)-induced C-X-C motif chemokine ligand 10 (CXCL10) secretion and decreased markers of healthy podocytes, suggesting a role in podocyte injury and inflammatory tissue remodeling characteristic of lupus nephritis (Weinstein et al, 2021).

The discovery of a previously unrecognized proteolytic fragment of C3 with noncanonical activities is both conceptually and therapeutically significant. Complement dysregulation contributes to a broad range of human pathologies and thus represents an increasingly important therapeutic focus (West et al, 2024). The modular nature of the complement network, with its distinct activation routes and effector arms, offers multiple nodes for precise, disease-specific intervention. Yet, accumulating insights into the biology of complement has revealed layers of complexity that challenge drug development. For example, complement operates not only systemically but also at tissue, cell-surface, and intracellular levels; it participates in immune homeostasis; and its components perform unexpected noncanonical functions (West and Kemper, 2023). Moreover, complement is not a simple top-down cascade, but a tightly regulated proteolytic network or web endowed with feedback and cross-regulatory loops (Mastellos et al, 2023). The inhibitory activity of C3-LHF1 on the lectin and classical pathways exemplifies such feedback control. Notably, while Demir et al, (2025) confirmed C3 as a substrate for MASP1/3, the cleavage site at position 1514 is also accessible in iC3b, raising the question of which C3 forms are sources of C3-LHF1 in vivo, under what conditions, and how this proteolytic process is regulated. Defining the molecular mechanism by which C3-LHF1 interferes with complement activation will be essential for future pharmacological manipulation of its generation or function. Likewise, the fragment’s apparent ability to modulate IL6ST signaling warrants dedicated investigation to establish its physiological relevance and therapeutic potential. Together, these findings expand the repertoire of known complement-derived effectors and reinforce the view that C3 biology is far from fully explored (West and Kemper, 2023). The unique dataset generated by Demir et al, (2025) further revealed unexpected N termini changes in complement components C4 and C5, offering additional avenues for investigation into their clinical significance. Another noteworthy observation was the marked divergence between plasma N-terminome and bulk proteome data, particularly for complement proteins. Standard proteomic assays, such as Olink, measure total protein abundance but do not capture activation states (Kirsher et al, 2025). As demonstrated in this study, combining high-throughput, ultra-sensitive, and complementary proteomic techniques provides a more complete view of proteolytic activation dynamics across disease states.

In summary, the work by Demir et al, (2025) delivers a comprehensive public resource of the healthy and autoimmune human plasma N-terminome and identifies a novel bioactive complement C3 fragment with dual roles in immune regulation and tissue pathology. Together, these discoveries advance our mechanistic understanding of lupus and highlight the potential of N terminomics for biomarker discovery and therapeutic innovation across immune-mediated diseases.

## References

[CR1] Demir F, Kovalenko E, Lassé M, Svenningsen E, Jensen JMB, Billing AM, Groeneveld K, Hutzfeldt A, Nilges L, Guerra JPL et al (2025) Proteolytic profiling of human plasma reveals an immunoactive complement C3 fragment. EMBO J. 10.1038/s44318-025-00598-8 10.1038/s44318-025-00598-8 PMC12706077 41145914

[CR2] Janssen BJ, Huizinga EG, Raaijmakers HC, Roos A, Daha MR, Nilsson-Ekdahl K, Nilsson B, Gros P (2005) Structures of complement component C3 provide insights into the function and evolution of immunity. Nature 437:505–511 16177781 10.1038/nature04005

[CR3] Kirsher DY, Chand S, Phong A, Nguyen B, Szoke BG, Ahadi S (2025) Current landscape of plasma proteomics from technical innovations to biological insights and biomarker discovery. Commun Chem 8:279 40999057 10.1038/s42004-025-01665-1 PMC12462477

[CR4] Klein T, Eckhard U, Dufour A, Solis N, Overall CM (2018) Proteolytic cleavage-mechanisms, function, and “omic” approaches for a near-ubiquitous posttranslational modification. Chem Rev 118:1137–1168 29265812 10.1021/acs.chemrev.7b00120

[CR5] Mastellos DC, Hajishengallis G, Lambris JD (2023) A guide to complement biology, pathology and therapeutic opportunity. Nat Rev Immunol 24:118–141 37670180 10.1038/s41577-023-00926-1

[CR6] Osman EEA, Neamati N (2024) Ironing out the mechanism of gp130 signaling. Pharmacol Rev 76:1399–1443 39414364 10.1124/pharmrev.124.001245

[CR7] Uzozie AC, Tsui J, Lange PF (2022) HUNTER: sensitive automated characterization of proteolytic systems by N termini enrichment from microscale specimen. Methods 2456:95–122 10.1007/978-1-0716-2124-0_8 35612738

[CR8] Weinstein A, Alexander RV, Zack DJ (2021) A review of complement activation in SLE. Curr Rheumatol Rep 23:16 33569681 10.1007/s11926-021-00984-1 PMC7875837

[CR9] West EE, Kemper C (2023) Complosome - the intracellular complement system. Nat Rev Nephrol 19:426–439 37055581 10.1038/s41581-023-00704-1 PMC10100629

[CR10] West EE, Woodruff T, Fremeaux-Bacchi V, Kemper C (2024) Complement in human disease: approved and up-and-coming therapeutics. Lancet 403:392–405 37979593 10.1016/S0140-6736(23)01524-6 PMC10872502

